# Glomerulonephritis in AKI: From Pathogenesis to Therapeutic Intervention

**DOI:** 10.3389/fmed.2020.582272

**Published:** 2021-03-02

**Authors:** Francesco Pesce, Emma D. Stea, Michele Rossini, Marco Fiorentino, Fausta Piancone, Barbara Infante, Giovanni Stallone, Giuseppe Castellano, Loreto Gesualdo

**Affiliations:** ^1^Nephrology, Dialysis and Transplantation Unit, Department of Emergency and Organ Transplantation, University of Bari, Bari, Italy; ^2^Nephrology, Dialysis and Transplantation Unit, Department of Medical and Surgical Science, University of Foggia, Foggia, Italy

**Keywords:** AKI, glomerulonephritis, antibodies, complement, immunosuppression

## Abstract

Acute kidney injury (AKI) is increasingly emerging as a global emergency. Sepsis, major surgery, and nephrotoxic drugs are the main causes of AKI in hospitalized patients. However, glomerulonephritis accounts for about 10% of AKI episodes in adults, mainly related to rapidly progressive glomerulonephritis resulting from granulomatous polyangiitis (GPA, Wegener granulomatosis), microscopic polyangiitis (MPA), and anti-glomerular basement membrane (GBM) disease. Also, diffuse proliferative lupus nephritis, immunoglobulin A nephropathy, post-streptococcal glomerulonephritis, mixed cryoglobulinemia, mesangiocapillary glomerulonephritis, membranous nephropathy, hemolytic uremic syndrome, thrombotic thrombocytopenic purpura, and scleroderma can induce acute renal failure. Early diagnosis of AKI due to glomerulonephritis is crucial for prompt, effective management to improve short- and long-term outcomes. Kidney biopsy is the gold standard for the diagnosis of glomerular disease, but it is not frequently performed in critically ill patients because of their clinical conditions. In this setting, a growing number of diagnostic assays can support the working hypothesis, including antineutrophil cytoplasmic antibodies (ANCAs), anti-double-stranded DNA antibodies, anti-GBM antibodies, antistreptolysin O and anti-DNase B antibodies, cryoglobulins, antiphospholipid antibodies, and complement levels. Therapeutic strategies in AKI patients with glomerulonephritis include high-dose corticosteroids, cyclophosphamide, and plasma exchange. This article reviews the wide spectrum of glomerulopathies associated with AKI, describing the immunological mechanisms underlying glomerular diseases and presenting an overview of the therapeutic options.

## Introduction

Acute kidney injury (AKI) is a severe medical condition involving up to 10 million people worldwide (1), with an increasing global incidence especially in hospitalized patients (2). AKI affects ~10–15% of hospital inpatients and more than 50% of patients hospitalized in the intensive care units (ICUs) (2, 3). Renal replacement therapy (RRT) is necessary in 5–6% of critically ill patients and is characterized by an increased risk of progression to chronic kidney disease (CKD) and end-stage kidney disease (ESKD) (about 10% annually) (4–6). The definition of AKI is based on standard criteria such as serum creatinine and/or urine output (7, 8), although several biomarkers have recently been proposed in this clinical setting (9). Severe short- and long-term consequences are frequently associated with AKI, and the mortality rate in critically ill patients is still significant, ranging from 37% to 60% (4, 10–12). Moreover, patients who survive AKI present a major long-term mortality rate and an increased risk of developing CKD (13) and other chronic comorbidities (14–19).

Glomerulonephritis accounts for about 10% of AKI in adults (20). AKI episodes in glomerular disease are usually due to rapidly progressive glomerulonephritis (RPGN), in which the renal function declines over days or weeks. The most common causes are small-vessel vasculitis and anti-glomerular basement membrane (GBM) disease, although other glomerular diseases may manifest with acute renal impairment, including IgA nephropathy (IgAN), thrombotic microangiopathy (TMA), lupus nephritis, and post-streptococcal glomerulonephritis (16). Furthermore, acute renal failure in glomerulonephritis can also result from non-glomerular conditions such as acute tubular necrosis (ATN) from renal hypoperfusion or the nephrotic syndrome and drug- or radiocontrast agent-induced tubular epithelial cell injury.

Early diagnosis and prompt, effective treatment of glomerular disease may dramatically change the disease course and improve patient outcomes (21). In this scenario, kidney biopsy remains the gold standard for the diagnosis of kidney disease when the patient's clinical condition allows the performance of this procedure (22). This article reviews the main glomerular diseases manifesting with AKI (summarized in Table 1), describing the immunological mechanisms underlying glomerular diseases and the potential therapeutic strategies, summarizing the main features.

**Table 1:** Summary of key features for each disease.

**Disease**	**Specific mechanisms of acute damage**	**Diagnosis**	**Kidney biopsy**	**Treatment**	**Apheresis**	**Prognostic markers**
Anti- GBM disease	Autoantibody against the NC1 domain of the α3-chain of type IV collagen (46, 47)	Clinical signs Anti-GBM antibody CT Kidney/lung biopsy	Diffuse proliferative GN, crescents, necrosis ± tubular loss IF: Linear deposition of IgG and C3	CYC (2 mg/kg/day) + Methylprednisolone (1 g/day for 3 days followed by tapering with oral prednisone) ± TPE	**TPE (Category III, Grade 2B)** Volume treated: 1–1.5 TPV Frequency: Daily or every other day Duration: at least 10–20 days, or until resolution of evidence of ongoing glomerular or pulmonary injury	Glomerular lesions: - crescents necrosis - Tubular damage Dialysis dependent sCr > 600 μmol/L
Small Vessel Vasculitis	ANCAs (PR3; MPO) and Neutrophils activation	Clinical signs ANCA antibody Radiological investigations Tissues biopsies/Kidney biopsy	Necrotizing and extra-capillary crescentic GN Vascular segmental fibrinoid necrosis, sclerosis, thrombosis	**Induction therapy:** Methylprednisolone (7 mg/Kg/day for 3 days followed by oral prednisolone 1 mg/Kg/day) + CYC (2 mg/kg/day) or, in alternative, RTX (375 mg/m^2^ weekly for 4 weeks) **Maintenance therapy:** AZA (1–2 mg/kg/day) or MMF (up to 1 g twice daily) or MTX (0.3 mg/kg/wk, maximum 25 mg/wk) or RTX	**TPE (Category I, Grade 1A)** Volume treated: 1–1.5 TPV Frequency: Daily or every other day Duration: median number is 7, over a median period of 14 days, up to 12 for further improvement of renal function BVAS	Glomerular lesions: - focal - crescents - mixed - sclerotic
SLE: lupus nephritis class IV	Immunocomplexes, complement, leucocytes, TMA	Kidney biopsy	Endocapillary or extracapillary GN, glomerulosclerosis, sub-endothelial immune deposits, tubular atrophy, interstitial fibrosis IF: “full house” pattern	**Induction therapy:** CYC (low-dose: 500 mg every 2 weeks for 3 months or high-dose: 0.5–1 g/m^2^ monthly for 6 months)/MMF (2–3 g/daily) + Corticosteroids. In alternative, RTX (375 mg/m^2^ weekly for 4 weeks) **Maintenance therapy**: Low dose Prednisone (<10 mg/day) + AZA (1.5–2 mg/kg/day) or MMF (1–2 g/day in two doses) for at least 12 months after complete remission. CNIs (in patients intolerant to MMF or AZA)	**TPE (Category II, Grade 2C)** Volume treated: 1–1.5 TPV Frequency: LN or DAH: daily or every other day; Other severe complications: 1–3 times per week Duration: course of 3–6 TPE	Glomerular lesions: - crescents - necrosis Heavy proteinuria Racial background
IgAN	Massive hematuria; erytro-phagocytosis process, crescents	Kidney biopsy	Mesangial hypercellularity, focal segmental necrotizing GN, crescents, glomerulosclerosis, tubule-interstitial inflammation ± fibrosis	High-dose corticosteroids (Methylprednisolone 1 g/daily for three consecutive days, followed by oral prednisone 0.5 mg/kg/day and subsequent tapering/Oral prednisone 1 mg/kg/day for 2 months followed by tapering) for 6 months + CYC (1–2 mg/kg/day) for 3 months, followed by AZA (1–2 mg/kg/day) for 3 months	**TPE (Category III, Grade 2C)** Volume treated: 1–1.5 TPV Frequency: 4–11 over 21 days Duration: until clinical resolution	High sCr Crescents Duration of hematuria
Post-streptococcal GN	Immunocomplexes, complement, leucocytes	Clinical signs Antistreptolysin O and anti–DNase B antibodies	Diffuse endocapillary GN, monocytes and lymphocytes infiltration	No specific immunosuppressive treatment	**TPE (Category III, Grade 2B)** Volume treated: 1–1.5 TPV Frequency: Daily or every other day Duration: 3–6 TPE over 1–2 weeks	Deficiency of complement regulatory proteins Nephrotic proteinuria Age Diabetic nephropathy
TMA	TMA	**TTP:** ADAMTS 13 activity, Anti ADAMTS13 autoantibody **STEC-HUS:** STEC (cultured test) or STX (P.C.R.) **aHUS:** microangiopathic haemolytic anemia, acute kidney injury, ADAMTS13 and STEC-HUS negativity	Intraluminal platelet thrombi, partial or complete obstruction of vessel lumina, myointimal proliferation and reduplication of the lamina densa, sever ischemic change	**TTP:** ADAMTS 13 auto-antibodies: TPE +/prednisone/prednisolone or RTX **Maintenance therapy:** PI: Caplacizumab + TPE + immunosuppressive therapy **STEC-HUS**: i.v 10–15 ml/kg/h of isotonic solution; Eculizumab (900 mg/ weakly for 4 weeks, followed by 1,200 mg every 2 weeks) and/or TPE/PI **aHUS:** Eculizumab (900 mg/ weakly for 4 weeks, followed by 1,200 mg every 2 weeks) **FHAA-mediated aHUS:** TPE + immunosuppressive therapy	**TPE (Category III, Grade 2C)** Volume treated: 1–1.5 TPV Frequency: Daily Duration: No standardized approach, duration and schedule should be made based upon patient response and condition	**TTP:** ADAMTS 13 activity; severe neurological signs; haemolysis **STEC-HUS:** severe neurological signs; haemolysis **aHUS:** genetic background
MPGN and Mixed Cryoglobulinemia	Immunocomplexes	Cryoglobulins, HCV markers; clinical signs/ symptoms	Mesangial and endocapillary proliferation, extracellular matrix expansion, diffuse leucocytes and monocytes infiltration, double-contouring of the GBM, intraluminal thrombi	TPE + RTX(375 mg7m^2^/week for 4 weeks)/CYC (2 mg/Kg/day for 2–4 months), + i.v. methylprednisolone (0.5/1 g/Kg/day for three consecutive days followed by oral prednisone), + antiviral therapy	**TPE (Category II, Grade 2B)** Volume treated: 1–1.5 TPV Frequency: Every 1–3 days Duration: 3–8 procedures for acute symptoms, weekly or monthly maintenance treatments to prevent recurrent symptoms	High sCr Nephrotic proteinuria Severe hypertension >50% crescents Marked interstitial fibrosis
Membranous nephropathy	Volume depletion Ischemic tubular damage	Kidney biopsy	Uniform increase in thickness of glomerular capillary walls, double-contouring and “spikes” of the GBM. IF: Diffuse, finely granular deposition of IgG along outer surface of all capillary walls	Volume correction	–	Severe hypoalbuminemia, age, male sex
Scleroderma	Ischemia, microangiopathy	Clinical signs; ANA and anti scl-70 antibody	Malignant hypertensions lesions, TMA in small interlobular and arcuate arteries Glomerular ischemic collapse; fibrinoid necrosis ± tubular atrophy and interstitial fibrosis	ACE-i TPE/ECP	**TPE (Category III, Grade 2C)** Volume treated: 1–1.5 TPV Frequency: 1–3 per week Duration: course of 6 TPE over the 2–3 weeks followed by 4 weekly treatments. Long-term TPE protocol (2–3 weekly for 2 weeks, 1 TPE weekly for 3 months, and 1 TPE every other week as a maintenance therapy) was also used. **ECP** Volume treated: Typically, 1.5 L of whole blood processed Frequency and Duration: Two procedures on consecutive days (one series) every 4–6 wk for 6–12 months	Anti-DNA polymerase

*GBM, Glomerular Basement Membrane; CT, Computer Tomography; GN, glomerulonephritis; IF, immunofluorescence; CYC, cyclophosphamide; TPE, therapeutic plasma exchange; sCr, serum creatinine; ANCAs, anti-neutrophil cytoplasmic autoantibodies, PR3, proteinase 3; MPO, myeloperosidase; RTX, Rituximab; AZA, azathioprine; MMF, mycophenolate; MTX, methotrexate; BVAS, Birmingham Vasculitis Activity Score; SLE, Systemic Lupus Erythematosus; TMA, Thrombotic Microangiopathy; IgA N, IgA nephropathy; TTP, thrombotic thrombocytopenic purpura; STEC-HUS, Shiga-toxin producing E. coli-associated Hemolytic Uremic Syndrome; STEX, Shiga-toxin; PCR, polymerase chain reaction; aHUS: atypical Hemolytic Uremic Syndrome; PI, Plasma Infusion; i.v., intravenous; FHAA-HUS, Factor H antibody associated Hemolytic Uremic Syndrome; MPGN, Membranoproliferative Glomerulonephritis; ANA, Anti-nuclear antibody; anti scl-70, anti-topoisomerase antibody, ACE-i, ACE inhibitors; ECP, Extracorporeal photopheresis*.

## Anti-GBM Disease

### Epidemiology and Disease Pathogenesis

Anti-GBM disease is a rare but severe immunological disorder manifesting with RPGN and pulmonary complications (lung hemorrhage). It accounts for about 5% of all cases of RPGN and affects especially young males and the elderly (23, 24). A genetic predisposition has been reported: anti-GBM disease is strongly associated with class II human leukocyte antigen (HLA), including DRB1^*^1501 and DR4 alleles, whereas DR1 and DR7 confer strong protection (25). Furthermore, several environmental triggers (smoking, hydrocarbon exposure, and drugs) appear to be important in the disease etiology (24, 26). The pathogenesis of the disease is immunological and related to the formation of antibodies specifically targeting the NC1 domain of the α3-chain of type IV collagen localized in the glomerular and alveolar basement membranes (24, 27, 28): complement activation, phagocyte accumulation, and T-cell recruitment after immunocomplex deposition contribute to the extent of the glomerular damage (29).

### Clinical Presentations and Diagnosis

Patients typically present a mild prodromal phase followed by severe clinical features with macroscopic hematuria and/or AKI. Lung involvement is often characterized by hemoptysis and dyspnea that characterize the Goodpasture syndrome. Kidney and respiratory functions decline more rapidly than in any other form of RPGN, and mortality is often due to renal failure requiring RRT or to massive alveolar bleeding (30). The clinical suspicion, based on the simultaneous renal and pulmonary involvement, is a critical step to improve patient outcomes. The rapid renal function decline, the presence of a very active urinary sediment, and the scarce systemic involvement differentiate anti-GBM disease from vasculitis and lupus nephritis. An atypical variant of the disease, with no significant pulmonary involvement and undetectable serum anti-GBM antibodies, has also been described (30). Antineutrophil cytoplasmic antibody (ANCA) can be detected in rare cases showing double antibody positivity. This double-positive variant presents a bad prognosis requiring early and more aggressive treatment (30).

The diagnosis is confirmed by the detection of circulating anti-GBM antibodies, although their levels do not correlate with disease severity (27). Kidney biopsy is essential as it indicates the extent and severity of the renal lesions. The histological picture in anti-GBM disease is usually characterized by diffuse extracapillary proliferation with extensive crescent formation, often with associated fibrinoid necrosis of the glomerular tuft. Different degrees of glomerulosclerosis (as a result of previous proliferative lesions), tubular necrosis, and interstitial inflammation can also be observed. A pathognomonic sign of the disease is the linear deposition of IgG and, often, C3 (75%) along the glomerular and sometimes (10–67%) also distal tubular basement membranes and the alveolar basal membrane in the lung. The extent of glomerular involvement (percentage of crescents and fibrinoid necrosis) is correlated with the prognosis (29, 30).

### Treatment

Since the clinical course is rapidly progressive, early treatment is needed, to remove circulating anti-GBM antibodies and reduce renal inflammation. Immunosuppressive therapy should be started immediately, except in patients with minimal renal involvement or irreversible kidney disease without lung involvement (31). The Kidney Disease Improving Global Outcomes (KDIGO) clinical practice guidelines for glomerulonephritis recommend the use of corticosteroids (usually intravenous pulses of methylprednisolone up to 1,000 mg/day for three consecutive days followed by oral prednisone of 1 mg/kg/day tapered to 20 mg/day for 6 weeks), oral cyclophosphamide (CYC, 2 mg/kg/day), and plasmapheresis (daily exchange of 1–1.5 volume of plasma against 5% human albumin for 14 days) in patients affected by anti-GBM disease (31–33). Lung hemorrhage is usually responsive to this treatment, and hemoptysis resolves within a few days. Renal recovery is more variable, and the clinical response to therapy is usually slower in patients requiring RRT and/or with serum creatinine values exceeding 600 μmol/L and in patients with histological evidence of a high proportion of crescents (34, 35). Oral prednisone and CYC are generally discontinued after 6 and 3 months, respectively, while plasmapheresis is continued for at least 14 days or until anti-GBM antibodies become undetectable.

## Small-Vessel Vasculitis

### Epidemiology and Disease Pathogenesis

Small-vessel vasculitis is a group of inflammatory systemic diseases characterized by a segmental necrotizing polyangiitis of small vessels, including GPA (Wegener granulomatosis), microscopic polyangiitis (MPA), eosinophilic granulomatosis with polyangiitis (Churg–Strauss syndrome), and renal-limited vasculitis (36). The first two diseases account for the majority of cases of RPGN, presenting as an emergency in the critical care setting (37, 38). The diseases can occur at any age, but the prevalence is greatest at ages 50–70 years, with a slight predominance in Caucasians and male gender (36). Small-vessel vasculitis is associated with the production of ANCAs, which have a central role in the disease pathogenesis, since they are directed against specific enzymes found within the cytoplasmic granules of neutrophils and monocytes, proteinase 3 (PR3), and myeloperoxidase (MPO) antigens (39, 40). Genetic background (specific α1-antitrypsin and neutrophil FcRγ111 receptor genotypes) and environmental triggers (in particular, infections and drugs) are reported as potential co-players in disease pathogenesis (41–45); specific HLA polymorphisms are associated with disease predisposition (46). After expression of PR3 and MPO on the surface following trigger conditions (infections), ANCAs bind these antigens, inducing neutrophil degranulation and the release of inflammatory mediators (cytokines, reactive oxygen species, and lytic enzymes), leading to endothelial damage (47, 48). Neutrophils also release PR3 and MPO, which adhere to the endothelium and induce *in situ* immunocomplex formation. Monocytes/macrophages, T cells, and complement system are also involved in the pathogenic process (49–52).

### Clinical Presentations and Diagnosis

Wegener granulomatosis is characterized by a wide range of clinical manifestations, including RPGN with the formation of extracapillary crescents, alveolar hemorrhage, episcleritis, rhinitis, sinusitis, hearing loss, purpura, peripheral neuropathy, subglottic tracheal stenosis, and angina abdominis (53). The disease course is often recurrent, with relapses occurring within a few years after disease remission. In patients with MPA, renal involvement is always reported, while respiratory tract diseases are less common; moreover, the frequency of relapses is lower than that in Wegener granulomatosis.

The clinical manifestations drive the diagnosis, which is supported by the detection of circulating ANCA. Anti-PR3 autoantibodies [cytoplasmic ANCA (c-ANCA)] are positive in about 90% of patients with active Wegener granulomatosis, and anti-MPO autoantibodies [perinuclear ANCA (p-ANCA)] are typically detected in about 80% of patients with active MPA (54, 55). Typically, ANCA levels are higher at the onset, and their levels are directly correlated with disease activity; in fact, a significant increase in ANCA levels is reported in relapses. These antibodies are also found in other immunological disorders (inflammatory bowel disease, autoimmune liver disease, and rheumatoid arthritis). A limited percentage of patients (10–20%) affected by small-vessel vasculitis does not show ANCA positivity (56). Thus, ANCA negativity may not exclude the diagnosis in the presence of clinical symptoms. Radiological investigations and tissue biopsies can support the diagnosis.

Although kidney biopsy in these patients is considered to be at high risk, since bleeding complications are frequent due to vessel inflammation, histology can be very useful for predicting the response to treatment and clinical outcome.

ANCA-associated glomerulonephritis is histologically characterized by necrotizing and crescentic glomerulonephritis, with a variable degree of glomerular involvement (57). Granulomas are a specific feature of Wegener granulomatosis (58, 59). Immunofluorescence shows little or no glomerular staining for immunoglobulins or complement, and this feature is typically termed a “pauci-immune” staining pattern. Electron microscopy demonstrates subendothelial edema, microthrombosis, and degranulation of neutrophils, without immune deposits (60). Vascular lesions feature fibrinoid necrosis, sclerosis, and thrombosis. Apart from glomerular and vascular lesions, acute and chronic tubulointerstitial lesions are critical risk factors indicating disease severity and progression. A recent histological classification comprises four categories of glomerular lesions, which correlate the loss of function with increasing degrees of severity: focal, crescentic, mixed, and sclerotic. Kidney survival at 5 years is 93% in the focal, 76% in the crescentic, 61% the mixed, and 50% in the sclerotic class (55). A growing number of studies have investigated the utility of TIMP 1, C–X–C motif chemokine ligand 13, and matrix metalloproteinase-3 as biomarkers of ANCA-associated vasculitis and for treatment assessment (61, 62).

### Treatment

A prompt diagnosis and early, appropriate treatment are essential to prevent loss of renal function and progression to CKD. Kidney and patient survival rates range between 65 and 75% at 5 years after appropriate treatment (63). KDIGO guidelines recommend the use of high-dose corticosteroids (intravenous methylprednisolone ar 7 mg/kg/day for 3 days followed by oral prednisone at 1 or 1 mg/kg/day of oral prednisone tapered to 0.25 mg/day for 3 months) plus oral or intravenous CYC (2 mg/kg/day or 0.5 g/m^2^ monthly, respectively. Combined treatment induces remission in about 90% of patients at 6 months. When CYC is contraindicated or the disease is resistant to this regime, the anti-CD20 monoclonal antibody rituximab (RTX) (375 mg/m^2^ weekly for 4 weeks) plus steroids is recommended (27–29). In this context, the RAVE and RITUXIVAS studies have shown that RTX is equivalent to CYC in terms of efficacy, with a similar risk profile (64, 65).

The addition of plasma exchange is recommended for patients requiring dialysis, with rapid increases of serum creatinine, diffuse pulmonary hemorrhage, and/or with an overlap syndrome of ANCA vasculitis and anti-GBM disease (31–33). Therapeutic plasma exchange (TPE) reduces the development of ESKD by ~40% (66), but the true efficacy is still questioned (67).

During maintenance therapy, CYC is replaced by azathioprine (AZA, 1–2 mg/kg/day) or alternatively mycophenolate (MMF, up to 1 g twice daily) or methotrexate (initially 0.3 mg/kg/week, maximum 25 mg/week). An alternative regimen includes the use of CYC for the induction treatment and perhaps RTX for maintenance (32, 33). Patients should be monitored for general symptoms and laboratory data, as well as urinary RBC counts, C-reactive protein, renal function, and ANCA levels. A rapid response to treatment is typical, with a significant improvement of the clinical condition within few weeks. High ANCA levels may persist for several months. The maintenance treatment is carried out for at least 18 months up to 3 years in Wegener granulomatosis and 2 years in MPA if no relapses occur (68), which can often happen during immunosuppression tapering and may require a further short course of high-dose corticosteroids or CYC.

In ANCA-associated pauci-immune glomerulonephritis, the mortality rate within the first year of diagnosis is reported to be about 20% of cases: ESKD occurs in up to 25% of the patients within 4 years after diagnosis (63).

## Other Glomerulonephritis Forms That Cause AKI

### Systemic Lupus Erythematosus (SLE)

Renal involvement in patients with SLE disease is associated with a worse outcome and higher mortality. Kidney damage is characterized by the deposition of immunocomplexes, complement activation, leukocyte infiltrations, and microangiopathic thrombosis probably driven by the type I interferon signature, involving all renal compartments (glomeruli, vessels, and tubule interstitium) (69). The histological findings in patients with lupus nephritis (LN) may range from initial mesangial immune deposits [class I in the World Health Organization (WHO) classification] to diffuse global glomerulosclerosis (class VI) (70); however, <10% of all patients with LN develop RPGN, which is associated with the diffuse proliferative glomerulonephritis form (Class IV).

Clinically, LN should be suspected in the presence of urinary abnormalities (hematuria) and nephrotic proteinuria; in addition, patients with Class IV LN typically exhibit acute impairment of renal function and hypertension. Low complement levels and positivity for anti-dsDNA antibodies may confirm the clinical suspicion. However, renal biopsy is crucial to assess the extent of renal lesions and to tailor appropriate treatment (21, 71, 72). In class IV LN, more than 50% of the glomeruli exhibit endocapillary hypercellularity and/or cellular crescents associated with other active (i.e., fibrinoid necrosis, karyorrhexis, presence of neutrophils, abundant subendothelial immunocomplex deposits, and interstitial inflammation) and chronic lesions (global and segmental glomerulosclerosis, adhesions, fibrous crescents, interstitial fibrosis, and tubular atrophy). Immunofluorescence shows a typical “full house” pattern, characterized by the evidence of IgG, IgM, IgA, C3, and C1q immune deposits. Electron microscopy reveals abundant electron-dense subendothelial deposits conferring a “wire loop” profile to the capillary walls (70, 73).

In patients with class IV LN, induction therapy can be based on intravenous CYC (low dose: 500 mg every 2 weeks for 3 months or high dose: 0.5–1 g/m^2^ monthly for 6 months) or MMF (2–3 g/day) combined with oral glucocorticoids (0.5–1.0 mg/kg/day) with or without three pulses of intravenous methylprednisolone (32, 33). In addition, RTX and other biological agents may be useful in class IV LN and in refractory LN as induction therapy (71). The induction therapy may be associated with TPE, as reported in Table 1 (67). After the induction treatment, maintenance therapy is required; oral AZA (1.5–2.5 mg/kg/day) or MMF (1–2 g/day in divided doses) is recommended and effective, combined with low-dose oral prednisone (≤10 mg/day) (31–33). The maintenance therapy should be continued for at least 1 year after complete remission is achieved, while a repeat kidney biopsy is required when complete remission has not been achieved. The maintenance phase of the Aspreva Lupus Management Study (ALMS) showed that regardless of the induction therapy, MMF was superior to AZA in maintaining the renal response and preventing relapse of proliferative LN: the overall treatment failure rate in the MMF group was half that observed in the AZA group (16.4 vs. 32.4%), and renal flares were significantly higher in patients treated with AZA (12.9 vs. 23.4%) (74). The superiority of MMF demonstrated in the ALMS was not apparent in the MAINTAIN nephritis trial, published in 2010 (probably due to differences in the study design): however, renal flares occurred in 19% of the MMF group, compared with 25% of the AZA group, suggesting that MMF should be considered the drug of choice (75). Finally, calcineurin inhibitors (CNIs) should be used for maintenance therapy in patients intolerant to MMF and AZA.

### IgAN

IgAN is the most common primary glomerulonephritis worldwide; it is characterized by persistent microscopic hematuria, mild proteinuria, and episodes of gross hematuria concurrently with upper respiratory tract infections (76). However, RPGN has been described in one third of patients with IgAN where histology reveals >50% crescents. Crescentic IgAN is a critical condition, leading to CKD and ESKD in a few years and so requiring prompt, efficacious treatment (77, 78). High-dose corticosteroids and CYC are recommended (79, 80). Corticosteroid treatment can be based on methylprednisolone (1 g/day for three consecutive days), followed by oral prednisone 0.5 mg/kg/day and subsequent tapering or, alternatively, on oral prednisone 1 mg/kg/day for 2 months followed by tapering (0.2 mg/kg monthly) within 6 months. CYC [1–2 mg/kg/day, based on the patient's glomerular filtration rate (GFR)] should be administered for 3 months, followed by AZA (1–2 mg/kg/day) for the following 3 months. TPE can be combined with immunosuppressive treatments until clinical improvement is achieved (67).

In addition, AKI episodes in IgAN patients can be due to massive hematuria of glomerular origin (81). In cases of massive hematuria, tubular cell damage has been described as the consequence of red blood cell cast formation and intratubular obstructions, combined with erythrophagocytosis processes (81). The hemoglobin products released by red blood cells induce oxidative stress, inflammation, podocytes, and tubular cell apoptosis and consequently cell detachment and fibrosis (82). In this setting, adequate and sustained hydration is crucial to prevent renal damage and enhance renal recovery. Renal biopsy should be performed in cases of persistent kidney function impairment despite supportive care and massive hydration.

### Post-streptococcal Glomerulonephritis

Post-streptococcal glomerulonephritis typically occurs in children in developing countries at 1–3 weeks after upper respiratory tract infections (83). Clinically, 20% of affected patients develop a classic nephritic syndrome characterized by hematuria, hypertension, oliguria, and worsening renal function. The diagnosis is based on the presence of these symptoms associated with the detection of antistreptolysin O and anti-DNase B antibodies and low C3 (83). In this setting, renal biopsy is not mandatory since the prognosis is usually excellent and no specific treatments are needed (21). However, 1% of patients develop crescentic glomerulonephritis, which is sometimes associated with ANCA (83). The role of immunosuppression in these patients is debated. Although there is no evidence yet from randomized controlled trials (RCTs), the use of high-dose corticosteroids can be considered in patients with extensive glomerular crescents and RPGN.

### TMA

TMA is a clinical phenotype which includes multiple diseases leading to thrombosis of arterioles and capillary vessels. Laboratory findings (thrombocytopenia with microangiopathic hemolytic anemia) lead to a suspicion of TMAs and reflect the mechanical disruption of red blood cells and platelets in the microvasculature (84). Thrombotic thrombocytopenic purpura (TTP) and hemolytic uremic syndrome (HUS) are the two classic TMA disorders (85). TTP is mainly characterized by neurological involvement, while only a limited proportion of patients (25%) show renal involvement; TTP patients usually exhibit congenital or acquired ADAMTS 13 deficiency leading to the accumulation of the ultra-large multimers of von Willebrand factors (vWFs) that cause platelet aggregation and consequently microvascular thrombosis (86). Conversely, kidney injury typically occurs in all patients with HUS, as a consequence of the massive thrombotic capillaries' occlusion (87). The histological features comprise microvascular thrombosis, swelling, and detachment of the endothelial cells from the GBM; patchy cortical necrosis; and focal-segmental sclerosis, which is a long-term sequela of acute HUS (88, 89).

HUS includes STEC-HUS due to Shiga toxin-producing *Escherichia coli* infection (90–92), pneumococcal HUS caused by infections with *Streptococcus pneumoniae*, and genetic HUS, also called aHUS (93). STEC-HUS occurs most frequently in pediatric patients (the incidence is five to six children per 10,000 children population per year). Patients typically present bloody diarrhea and gastroenteritis 6–10 days before the development of TMA. STX closely adheres to gut epithelial cells, causing apoptosis and destruction of the brush border of the villi. Then, the toxin enters the circulation: in the kidney, STX is internalized via the Gb3 receptor and releases a protease into the cytoplasm. This protease inhibits protein synthesis and activates inflammatory pathways, inducing cell death (92). The diagnosis is driven by the clinical suspicion and the detection of STEC (cultured test) or STEX (polymerase chain reaction). Prompt intravenous hydration (10–15 ml/kg/h of isotonic solution) is recommended as soon as *E. coli* infection is suspected, in order to reduce the systemic effects of STX (94, 95). The use of antibiotics must be avoided, because they increase STX gene expression and induce massive STX release. Only azithromycin can be administered to a very limited number of children presenting with bacteremia (94). Supportive management of anemia, renal failure, and hypertension is required in these patients. In cases of severe clinical manifestations and neurological involvement, eculizumab and/or plasma exchange can be used as rescue therapy (88). The prognosis in children is generally favorable, and up to 70–85% of patients recover renal function.

aHUS is a multifactorial rare disease linked to an uncontrolled activation of the alternative complement pathway. About 60% of aHUS patients carry genetic variants or risk haplotypes in genes of the alternative pathway (CFH, CFI, CFB, MCP, C3, CFHR1-5, and THBD) and/or in diacylglycerol kinase E (DGKE). Genetic background accounts for different outcomes, as response to therapy and risk of relapses may vary depending on the underlying mutation; in addition, specific environmental factors (pregnancy, drugs, infections, etc.) have been reported as triggers in disease pathogenesis (93). aHUS is a life-threatening disorder requiring immediate diagnosis and treatment, often in the ICU. In addition, 50% of aHUS patients progress to ESKD, and the mortality rate during the acute phase of the disease is significant. The diagnosis of aHUS is made on exclusion of STEC infection and ADAMTS 13 deficiency; moreover, a genetic screening should be recommended, particularly in patients who are candidates for kidney transplantation. Treatment should be promptly started. The use of eculizumab in the aHUS treatment has dramatically changed short- and long-term outcomes in this setting and is currently the first-line treatment. The attack dose is 900 mg weekly for 4 weeks, followed by 1,200 mg at week 5 and then every 2 weeks: however, the duration of treatment is not yet well established. The Food and Drug Administration and the European Medicines Agency have recently approved a long-acting anti-C5 antibody (ravulizumab) for the treatment of aHUS, to be administered every 8 weeks (half-life of approximately 51 days).

It is important to remember that patients with DGKE variants do not respond to eculizumab therapy and thus pose an open challenge for scientists and physicians (96). Finally, 3–8% of aHUS patients present antibodies to complement factor H protein (FHAA), which are associated with a homozygous deficiency of FHR1 (85%) and homozygous deletion of CFHR3-CFHR1 (97). In this setting, plasma exchange alone was associated with disease recurrence (58%), CKD (39%), ESKD (27%), and death (9%) (98). A combination of TPE and immunosuppression reduces the antibody production and improves patient outcomes (99, 100).

### Membranoproliferative Glomerulonephritis (MPGN) and Mixed Cryoglobulinemia

In MPGN, the histological pattern includes a group of chronic immune-mediated diseases characterized by GBM thickening and proliferative changes (101). On the basis of the pathophysiological processes, MPG is currently classified as immunocomplex-mediated MPGN and complement-mediated GNs (DDD and C3GN) (102). Among these disorders, mixed cryoglobulinemia is the most prevalent disorder in older adults (103). Mixed cryoglobulinemia is a small-vessel vasculitis characterized by the precipitation of circulating immunocomplexes after cold exposure (polyclonal IgG with or without monoclonal IgM with rheumatoid factor activity), involving several organs such as the skin, the joints, the peripheral nervous system, and the kidneys (103). Hepatitis C virus (HCV) disease is the main cause of mixed cryoglobulinemia. In HCV-positive patients, renal involvement accounts for about 30–40% of all cases (104). Clinical symptoms are weakness, arthralgias, and purpura, followed by a nephritic/nephrotic syndrome and rapidly deteriorating kidney function (104, 105). Kidney histology is characterized by mesangial and endocapillary proliferation, extracellular matrix expansion, diffuse leukocyte and monocyte infiltration, double contouring of the GBM, and the presence of intraluminal PAS-positive thrombi. On electron microscopy, electron-dense deposits (cryo-immunocomplexes) can be detected in subendothelial and mesangial regions (104, 106). The diagnosis is based on specific laboratory findings (detection of serum cryoglobulins and serologic hepatitis C markers) combined with the clinical signs and symptoms (104). In this scenario, KDIGO guidelines suggest the use of TPE combined with immunosuppressive treatment, such as RTX (375 mg/m^2^/week for 4 weeks) or CYC (2 mg/kg/day for 2–4 months), combined with intravenous methylprednisolone (0.5/1 g/kg/day for three consecutive days followed by oral prednisone). Importantly, concomitant antiviral therapy should be performed in HCV-positive patients (31–33).

### Glomerulonephritis Manifesting With Nephrotic Syndrome

Several forms of glomerulonephritis are associated with a nephrotic syndrome, characterized by heavy proteinuria, severe hypoalbuminemia, edema, weight gain due to fluid retention, and dyslipidemia. Membranous nephropathy is the main cause of nephrotic syndrome in adults (107); in addition, diabetic nephropathy, minimal change disease, focal-segmental glomerulosclerosis, SLE, and amyloidosis usually manifest with nephrotic syndrome. AKI episodes have been in rare cases described as a potential complication of nephrotic syndrome, independently of the underlying pathogenesis (108, 109). The AKI pathogenesis in this setting is multifactorial. Some clinical observations supported the hypothesis that the massive urinary albumin loss induces microvascular injury due to volume depletion (109), leading to a massive expression of endothelin 1, inducing a decrease in blood flow and GFR (110). In addition, ischemic tubular damage and cell necrosis have been reported in some cases. Recent studies have shown that some urine biomarkers (NGAL and alpha 1-microglobulin) are elevated in patients with AKI and nephrotic syndrome (111). Although the pathogenesis still needs to be clarified, AKI in NS is generally reversible. The majority of patients with idiopathic nephrotic syndrome have a complete recovery of kidney function after volume depletion correction. Severe hypoalbuminemia, older age, and male sex are risk factors (109).

### Scleroderma

Scleroderma is an autoimmune systemic disorder characterized by uncontrolled expansion of connective tissue in skins and other visceral organs. Kidney involvement is usually characterized by the presence of low-grade proteinuria, but AKI episodes can occur (112, 113). The so-called scleroderma renal crisis is a life-threatening complication of scleroderma and typically presents with the abrupt onset of hypertension and rapid progressive renal insufficiency, followed by encephalopathy, heart failure, and signs and symptoms of TMA (113, 114). The typical histological features are ischemic glomerular changes, malignant hypertension lesions, and TMA in small interlobular and arcuate arteries: glomeruli may show ischemic collapse and fibrin thrombi; various degrees of tubular atrophy and interstitial fibrosis have also been reported (115). Clinical presentation, along with the detection of speckled ANA (positive in 90% of cases) and anti-topoisomerase I antibodies (scl-70) (positive in 20% of cases), supports the diagnosis (113). Renal biopsy should be performed in patients with atypical presentations (increased serum creatinine in normotensive patients or in the presence of active urine sediment or nephrotic range proteinuria). Activation of the renin–angiotensin–aldosterone system (RAAS) is crucial in the pathogenesis of scleroderma renal crisis. Thus, the introduction of angiotensin-converting enzyme (ACE) inhibitors has substantially improved the prognosis of patients with scleroderma crisis, reducing the mortality associated with scleroderma crisis to <10% (113). Calcium channel blockers and, as third line, diuretics and alpha-blockers could be used as additional therapy if blood pressure control remains suboptimal despite maximum doses of ACE inhibitors (116). In addition, recent evidence suggests a role of complement activation and endothelin-1 in the pathogenesis of scleroderma crisis, thus suggesting the use of C5 inhibitors and endothelin receptor antagonists, particularly in refractory cases. Finally, TPE seems to confer some benefits in patients with scleroderma crisis with evidence of TMA or in patients with ACE inhibitor intolerance (116): a typical course of six TPE over 2–3 weeks followed by 4-weekly treatments is a reasonable therapeutic approach, resulting in long-lasting improvements in symptoms (67).

## Conclusions

Several glomerular diseases have been reported to manifest with AKI episodes: early diagnosis is crucial since different conditions have a similar clinical profile but require different, often aggressive, treatment in order to preserve renal function and delay the onset of ESKD. In this scenario, renal biopsy is essential for an accurate diagnosis and to describe the extent of reversible/irreversible renal lesions.

## Author Contributions

FPe and ES wrote the manuscript. MR provided histological images. MF, FPi, GS, GC, and LG revised the paper. All authors contributed to the article and approved the submitted version.

## Conflict of Interest

The authors declare that the research was conducted in the absence of any commercial or financial relationships that could be construed as a potential conflict of interest.
